# Lead enhances the phytotoxicity of *Aegilops tauschii* toward wheat seedlings

**DOI:** 10.3389/fpls.2025.1715391

**Published:** 2025-12-10

**Authors:** Ning Wang, Zixuan Huang, Xiaoxiao Shen, Yaowu Tian

**Affiliations:** College of Horticulture and Plant Protection, Henan University of Science and Technology, Luoyang, Henan, China

**Keywords:** *Aegilops tauschii*, allelopathic, antioxidant enzyme system, endogenous hormones, *Triticum aestivum*

## Abstract

Heavy metal pollution in agricultural soil poses a significant threat to food security and the health of the ecosystem. This study investigated the allelopathic effects of *Aegilops tauschii*, a common weed found in wheat fields, on winter wheat (*Triticum aestivum* L. cv. ‘Zhengmai 132’) under lead (Pb) contamination. A completely randomized factorial design was used, employing a Petri dish filter paper bioassay with three concentrations of *A. tauschii* stem-and-leaf extract (0, 25 and 50 g/L) and three Pb levels (20, 80 and 160 mg/L), with five replicates per treatment combination. Low and high concentrations of *A. tauschii* extract, as well as low Pb levels, had little effect on wheat germination or seedling growth when applied alone. In contrast, medium and high Pb concentrations significantly inhibited these parameters in a dose-dependent manner. Combining the extract with Pb resulted in quantitative analysis using Colby’s method confirmed a significant synergistic inhibition, causing pronounced suppression of germination and early growth (*P<* 0.05). Physiological and biochemical analyses revealed a strong, synergistic oxidative stress response when the extract of *A. tauschii* interacted with Pb. Although antioxidant enzymes (SOD, POD and CAT) were activated in an attempt to counteract the oxidative stress, this increase was insufficient to prevent cellular damage. Consequently, the oxidative burst intensified, resulting in membrane injury and a 38.95% increase in thiobarbituric acid-reactive substances (TBARS) under the SCHPbH treatment. The interaction also disrupted hormonal homeostasis, decreasing the levels of the growth-promoting hormones gibberellin (GA_3_), indole-acetic acid (IAA) and zeatin riboside (ZR), while increasing abscisic acid (ABA). Under the strongest combined treatment (SCHPbH), GA_3_ decreased by 37.7%, and ABA increased by 40.6%, leading to a 74.6% reduction in the GA_3_/ABA ratio (*P* < 0.05). These alterations to growth-promoting and -inhibiting signals significantly lowered the GA_3_/ABA and IAA/ABA ratios. In summary, Pb contamination greatly increases the allelopathic effect of *A. tauschii* on wheat. The strongest combined treatment inhibited germination and growth by around 48%, indicating severe physiological disruption. These results emphasise the significant threat that this interaction poses to the stability of agroecosystems.

## Introduction

Healthy soil is essential for sustainable agriculture, as its quality directly impacts food security (Long et al., 2024). However, the rapid growth of modern industry and farming has increased the release of harmful chemicals into the environment. Among these, heavy metals (HMs) are especially dangerous to land ecosystems (Iyyappan et al., 2023). A national risk assessment of HM contamination in three staple crops — rice (*Oryza sativa* L.), wheat (*Triticum aestivum* L.) and maize (*Zea mays* L.) — found that 26.77% of China’s cultivated land is at high risk, 60.89% is at medium risk and only 12.34% is in a safe condition (Zhou et al., 2024). National data on HM levels in rice and wheat show that the highest percentage of samples exceed the safe limit for lead (Pb) in wheat, followed by cadmium (Cd) (Wang et al., 2023). HM pollution not only affects seed germination and seedling growth (Jiang et al., 2019; Lei et al., 2021) but also impacts physiological and ecological traits (Sharma et al., 2022; Rashid et al., 2023), ultimately reducing crop yield and quality (Lu et al., 2018). Moreover, HM contamination can alter the physical and physiological characteristics of invasive plants in agricultural fields, increasing their competitiveness and invasion potential (Wei et al., 2020a; Yu et al., 2020), which compromises farmland ecosystem stability. Winter wheat (*Triticum aestivum* L.) is a vital crop worldwide, forming the backbone of agricultural production in China. However, vast areas of winter wheat cultivation face challenges from abiotic stresses, such as heavy metal contamination, and biotic pressures, such as invasive weeds (Zhou et al., 2024). This complex interplay has a significant impact on crop yield and food security. According to the ‘novel weapon hypothesis,’ invasive plants may gain an advantage by releasing allelochemicals toxic to native plants (Svensson et al., 2013). Studies show that HMs can influence the allelopathic effects of invasive species on crop seed germination and seedling growth, affecting their capacity to invade successfully (Wei et al., 2020b). For example, the absence of HM pollution has been shown to limit the spread of invasive species such as *Solidago canadensis* L (Yang et al., 2007a, b).

The study of allelochemicals is inherently complex. This is due to the wide range of variability in the compounds released by different weed species, and the difficulty of isolating their individual effects (Akter et al., 2023). However, existing research has demonstrated the important role of allelochemicals in the interaction between specific crops and weeds (Scavo and Mauromicale, 2021). For example, studies have reported allelopathic inhibition of maize germination by species such as *Amaranthus retroflexus* (Abbas et al., 2023) and of rice growth by *Setaria viridis* (Wang et al., 2020). Furthermore, research has demonstrated that heavy metal stress can modulate the allelopathic potential of certain weeds. For instance, studies have revealed that Pb contamination can amplify the release of phytotoxins from invasive plants, thereby magnifying their allelopathic impact on crops (Wei et al., 2020b). This finding aligns with the ‘novel weapon hypothesis’ (Svensson et al., 2013).

*Aegilops tauschii* Coss., belonging to the goatgrass genus within the Poaceae, is native to Eastern Europe and Western Asia (Zhao et al., 2020). As a highly invasive weed, it has rapidly spread across major wheat-producing provinces in China, including Henan, Shandong, and Shanxi, infesting wheat cultivation areas covering 330,000 hectares (Abbas et al., 2020). Its widespread infestation and evolution into a national issue (Qiang and Zhang, 2022) can be attributed to its high reproductive capacity, broad adaptability and ease of transmission (Fang, 2012), as well as its strong competitive ability (Wang and Chen, 2023; 2024), and phenotypic plasticity (Wang et al., 2019a). Although *A. tauschii* is recognised as a weed that releases allelochemicals which could affect crop growth, the exact nature and extent of these effects, particularly on winter wheat under heavy metal stress, is unclear. Inadequate preventive measures and limited control strategies have allowed *A. tauschii* to become a serious threat to wheat production security in China (Abbas et al., 2023). Despite its significant ecological and agricultural impact, research detailing the allelopathic mechanisms of *A. tauschii* specifically in relation to environmental stressors such as HM contamination, remains insufficient. To date, our research group is the first to demonstrate that *A. tauschii* exhibits strong adaptability to light and soil moisture stress by regulating allelochemical release and plasticity in biomass allocation (Wang et al., 2019b). However, the precise role of allelopathy in the interactions between *A. tauschii* and wheat, particularly in the context of agricultural soils polluted by heavy metals, is unclear. In view of the growing severity of HM contamination in agricultural soils and the potential for *A. tauschii* to behave as an invasive species in such environments, we hypothesise that: (H1) Pb contamination will enhance the phytotoxicity of *A. tauschii* towards wheat seedlings, resulting in more severe inhibition of seed germination and early seedling growth than either stressor alone. (H2) This phytotoxicity will be mediated by lead-induced disruption of wheat’s physiological and biochemical defence mechanisms, including the oxidative stress response and endogenous hormone balance. This study investigates the synergistic allelopathic effects of Pb pollution and *A. tauschii* extract on wheat seed germination and seedling growth, aiming to elucidate the underlying physiological and biochemical responses. Ultimately, our findings will shed light on the invasion mechanisms of *A. tauschii* in HM-contaminated farmland, providing a theoretical basis for ecological restoration and sustainable soil utilisation.

## Materials and methods

### Preparation of materials

The plant material was collected in December 2024 from an experimental field in Dianzhuang Town, Luolong District, Luoyang City, Henan Province, China (34°41’ N, 112°37’ E). Well-developed *A. tauschii* plants at the tillering stage were randomly selected before winter, and their above-ground tissues (stems and leaves) were harvested for subsequent analysis. The study region has a typical temperate monsoon climate. The mean annual temperature is 14.9°C, with an average annual precipitation of 578.2 mm. The area receives substantial solar radiation, enjoying 2,164 hours of sunshine per year, and benefits from an extended growing season of 219 frost-free days. The aerial parts of *A. tauschii* were sequentially rinsed with tap water and deionized water to remove surface contaminants. After air-drying, the plant material was cut into small pieces, and 50.0 g (± 0.0001 g) was accurately weighed and transferred to a 1-L beaker containing 500 mL of distilled water. The mixture was incubated at 25 °C for 48 h with occasional stirring. The aqueous extract was subsequently filtered through double-layer filter paper to remove plant debris, yielding a stock solution with a concentration of 100 g/L. The extract was stored at 4 °C until further use. Before use, the stock solution of *A. tauschii* aqueous extract was diluted with distilled water to obtain the following concentrations: (1) Control (CK): 0 g/L, representing non-invaded conditions; (2) Low concentration Solution of *A. tauschii* stem-leaf extracts (SCL): 25 g/L, simulating mild invasion; and (3) High concentration Solution of *A. tauschii* stem-leaf extracts (SCH): 50 g/L, simulating severe invasion. Pb solutions were prepared using lead acetate trihydrate (≥99.0% purity; Pb(CH_3_COO)_2_·3H_2_O; Shanghai Sangon Biotech Co., Ltd.). Based on reported levels of Pb contamination in Chinese soils (Zhou et al., 2024) and our previous findings regarding the effects of heavy metal stress on the growth of *A. tauschii* seedlings (Wang et al., 2024), three Pb concentrations were selected: 20, 80, and 160 mg/L.

### Experimental design

The experimental design consisted of nine treatments (**Table 1**), each of which had five independent biological replicates (n = 5). Each replicate was represented by a separate Petri dish. Uniform, fully developed seeds of wheat (*T. aestivum* cv. ‘Zhengmai 132’, provided by Luoyang Academy of Agricultural and Forestry Sciences) were surface-sterilized with 1% sodium hypochlorite for 5 min, followed by four rinses with distilled water. After blot-drying, 20 seeds were placed in each 10-cm Petri dish lined with double-layer filter paper. Single treatments received 10 mL of the corresponding solution, while combined treatments received 5 mL each of the extract and Pb solution. Petri dishes were incubated for 7 days in a growth chamber maintained at 20/15°C (day/night), with a 12-h photoperiod and 75% relative humidity.

**Table 1:** Summary of the experimental design in this study.

Abbreviation	Treatment	Concentration
CK	Control (distilled water)	0 g·L^-1^
SLC	Stem-leaf extracts with low concentration	25 g·L^-1^
SHC	Stem-leaf extracts with high concentration	50 g·L^-1^
PbL	Low Pb concentration	20
PbM	Medium Pb concentration	80
PbH	High Pb concentration	160
SCLPbL	The combined treatment of SLC and Pb with 20 mg·kg^-1^	The former with 25 g·L^-1^ and the latter with 20 mg·L^-1^
SCLPbM	The combined treatment of SLC and Pb with 80 mg·kg^-1^	The former with 25 g·L^-1^ and the latter with 80 mg·L^-1^
SCLPbH	The combined treatment of SLC and Pb with 160 mg·kg^-1^	The former with 25 g·L^-1^ and the latter with 160 mg·L^-1^
SCHPbL	The combined treatment of SHC and Pb with 20 mg·kg^-1^	The former with 50 g·L^-1^ and the latter with 20 mg·L^-1^
SCHPbM	The combined treatment of SHC and Pb with 80 mg·kg^-1^	The former with 50 g·L^-1^ and the latter with 80 mg·L^-1^
SCHPbH	The combined treatment of SHC and Pb with 160 mg·kg^-1^	The former with 50 g·L^-1^ and the latter with 160 mg·L^-1^

Based on temporal discrepancies in the initial metabolic responses and subsequent hormonal regulation observed during wheat seed germination in previous studies (Kucera et al., 2005; Li et al., 2024), this study involved sampling at specific time points. At 48 hours post-imbibition, seeds were harvested to quantify components of the antioxidant system, including superoxide dismutase (SOD; EC 1.15.1.1) and peroxidase (POD; EC 1.11.1.7) activities, as well as malondialdehyde (MDA) content, using a thiobarbituric acid reactive substances (TBARS) assay. Endogenous hormone levels were determined at 60 hours post-imbibition using an enzyme-linked immunosorbent assay (ELISA) on individual biological replicates. The hormone-containing filtrate fraction was processed as previously described (Wang et al., 2015). This involved dissolving the filtrate in 1 ml of phosphate-buffered saline (PBS), supplemented with 0.1% (v/v) Tween 20 and 0.1% (w/v) gelatin (pH 7.5). ELISA was performed using a commercially available kit (Cat. No. JLC-E2850, manufactured by Jianglan Pure, Jiangxi, China) in accordance with the manufacturer’s instructions. The assay targeted abscisic acid (ABA), indole-3-acetic acid (IAA), gibberellin A3 (GA_3_) and zeatin riboside (ZR). Germination dynamics were monitored daily using strict criteria: seeds were considered germinated once the radicle emerged.

### Index determination and method

Morphometric analyses at the end of the experiment included the following: (1) plant height (coleoptile length) and (2) root length (primary root), both measured with a centimetre ruler (accuracy: 0.10 cm); and (3) fresh weight (the combined weight of the shoot and root) measured using an electronic balance (accuracy: 0.0001 g). Three biological replicates were performed for each treatment.

SOD (EC 1.15.1.1) activity was quantified using a commercial assay kit (G0101F, Suzhou Geruisi Bio, China) based on the NBT photochemical reduction method at 560 nm (Li, 2000). POD (EC 1.11.1.7) activity was determined via guaiacol oxidation at 470 nm, with activity expressed as ΔA470 min^−1^ mg^−1^ protein (Li, 2000). CAT activity was assayed using the hydrogen peroxide decomposition method by measuring the decline in H_2_O_2_ absorbance at 240 nm. Lipid peroxidation levels were assessed through TBARS content, which was expressed as malondialdehyde (MDA) equivalents calculated based on the extinction coefficient of the MDA-TBA complex at 532 nm. All spectrophotometric measurements were conducted using analytical grade reagents on a 752N UV-Vis spectrophotometer (INESA Instrument Co., Ltd., Shanghai, China). Blank and standard controls were run alongside all samples to ensure accurate quantification.

The concentrations of GA_3_, ABA, IAA, and ZR were quantified using plant hormone-specific enzyme-linked immunosorbent assays (ELISA) for each respective plant hormone, according to the manufacturer’s protocols (Li, 2000). The GA_3_/ABA ratio was calculated as a critical physiological indicator of germination potential, reflecting the hormonal antagonism during the release of dormancy.

### Data analysis

The germination rate (GR) is calculated using the following formula:


GR= (Total number of seeds tested/Number of germinated seeds)×100

The response index (RI) was used to measure the stimulatory or inhibitory effect of each treatment on wheat seed germination and seedling growth (Ma et al., 2020). Following the convention of Williamson and Richardson (1988), the RI was calculated as follows:


$$RI=\{\begin{array}{c} \frac{T}{C}-1,if\;T<C\\ 1-\frac{C}{T},\;if\;T\geq C \end{array}$$

where C is the mean value of the control and T is the mean value of the treatment.

An RI greater than 0 indicates promotion, an RI of 0 denotes no significant allelopathic effect, and an RI less than 0 signifies inhibition.

### Assessment of interactive effects

The nature of the interaction between the *A. tauschii* extract and Pb was determined using Colby’s method (Colby, 1967). The expected inhibition (*E*) for each combined treatment was calculated using the following formula, assuming an additive effect:


$$E=X+Y-\frac{X\times Y}{100}$$

where *X* and *Y* represent the percentage inhibition of germination (or other growth parameters), caused by *A. tauschii* extract or Pb alone respectively, compared to the control. The observed inhibition (O) was obtained directly from the experimental data for the combined treatment.

The interaction was interpreted as follows:

Synergistic: Observed inhibition is significantly greater than the expected inhibition. (O > E).
Antagonistic: Observed inhibition is significantly less than the expected inhibition. (O< E).
Additive: Observed inhibition is not significantly different from expected inhibition. (*O* = *E*).

A paired t-test was used to compare the observed and expected values for each combined treatment, at a significance level of *P* <0.05.

All experimental data underwent rigorous statistical evaluation. Prior to analysis, the assumptions of normality and homoscedasticity (equality of variances) for the one-way analysis of variance (ANOVA) were verified. The Shapiro–Wilk test confirmed that the data did not significantly deviate from a normal distribution (*P* > 0.05) and Levene’s test confirmed homogeneity of variances across the treatment groups (*P* > 0.05). As all assumptions were met, a one-way ANOVA was employed to identify significant variations among the treatment groups. *Post-hoc* pairwise comparisons were performed using the Tukey honestly significant difference (HSD) test to determine which treatment combinations differed significantly in terms of germination rates, radicle emergence kinetics and seedling morphometric parameters (shoot height, root length and fresh biomass). The threshold for statistical significance was set at P ≤ 0.05. All analyses were conducted using IBM SPSS Statistics v26, and the results are expressed as mean values ± standard error in the accompanying figures.

## Results

**Figure 1:**
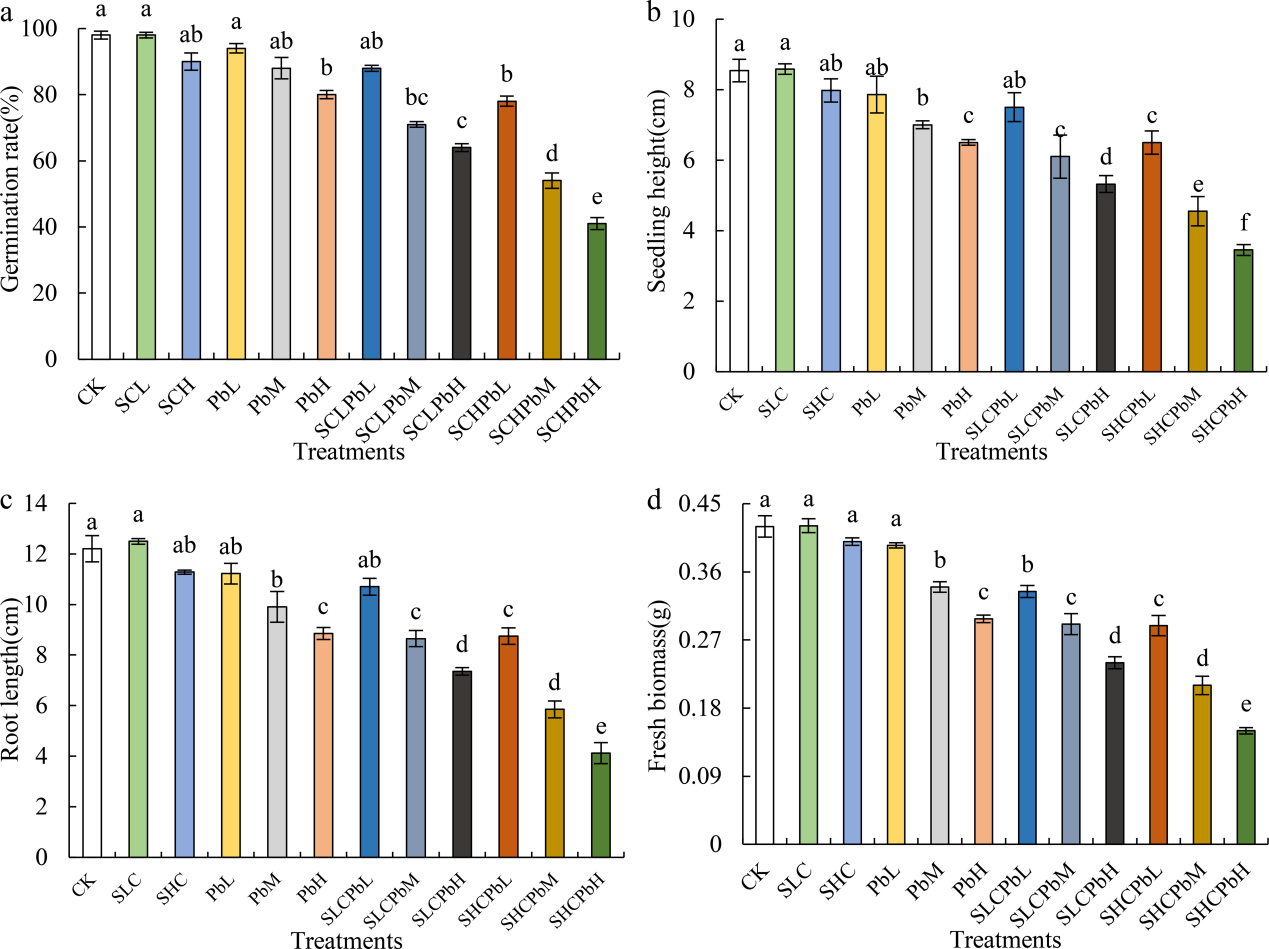
Seed germination and seedling growth parameters of wheat in different treatment groups. Bars (mean ± SE) with different lowercase letters indicate a significant difference (*P* < 0.05). **(a)** germination rate, **(b)** seedling height, **(c)** root length, and **(d)** fresh biomass. CK, control; SCL, *A. tauschii* stem-leaf extract (low concentration); SCH, *A. tauschii* stem-leaf extract (high concentration); PbL, low Pb concentration; PbM, medium Pb concentration; PbH, high Pb concentration; SCLPbL, SCL and PbL; SCLPbM, SCL and PbM; SCLPbH, SCL and PbH; SCHPbL, SCH and PbL; SCHPbM, SCH and PbM; SCHPbH, SCH and PbH.

**Figure 2:**
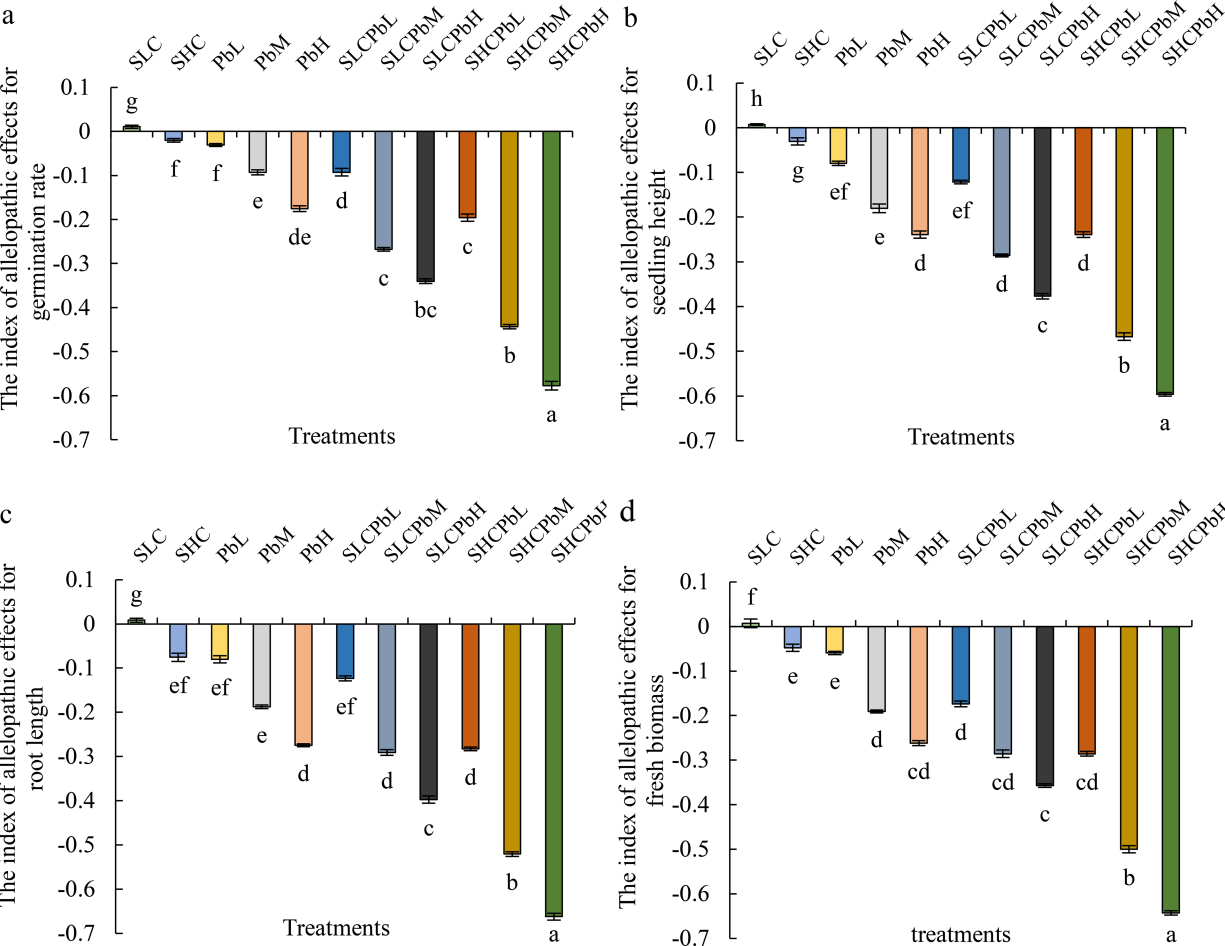
Indices of allelopathic effects on wheat germination parameters in different treatment groups (see **Figure 1** for abbreviations). Bars (mean ± SE) with different lowercase letters indicate a significant difference (*P* < 0.05). The index indicates a positive impact when the value is positive, and a negative impact when the value is negative. Index of allelopathic effects on **(a)** germination rate, **(b)** seeding height, **(c)** root length, **(d)** fresh biomass.

All treatments exhibited varying degrees of inhibition of wheat seed germination and early seedling growth (**Figure 1**). These treatments included stem-leaf extracts alone (SCL and SCH), lead (Pb) solutions alone (PbL, PbM and PbH) and combinations of these. In most cases, individual applications of the stem-leaf extracts and the low-concentration lead solution (PbL) resulted in only minor, statistically non-significant effects (**Figure 2**). By contrast, medium- and high-concentration Pb solutions (PbM and PbH) significantly reduced the germination rate, seedling height, root length and fresh biomass (*P* < 0.05). Combined treatments of stem-leaf extracts and Pb solutions demonstrated a clear synergistic inhibitory effect on wheat seed germination and early growth. As shown in **Figure 1, a** dose-dependent pattern was evident, with the intensity of inhibition increasing alongside rising Pb concentrations. The SCHPbH treatment had the strongest inhibitory effect, reducing the germination rate, seedling height, root length and fresh weight by 47.44%, 46.92%, 52.91% and 48.10% respectively compared to the control (*P* < 0.05).

The interaction between the *A. tauschii* extract and Pb was analysed quantitatively using Colby’s method (**Table 2**). All combined treatments exhibited significant synergistic inhibition of wheat seed germination, with the intensity of synergy increasing at higher concentrations. For instance, the observed inhibition (58.16%) in the SCHPbH treatment was over twice the predicted additive effect (25.03%) (*P* < 0.05), which clearly indicates a strong synergistic interaction.

**Table 2:** Effect of *A. tauschii* extract on wheat seed germination in the presence of Pb, as determined by Colby’s method.

Combined treatment	Inhibition by extract alone (X %)	Inhibition by Pb alone (Y %)	Expected inhibition (E %)	Observed inhibition (O %)	Interaction type
SCLPbL	0	4.08	4.08	10.20^*^	Synergistic
SCLPbM	0	10.20	10.20	27.55^*^	Synergistic
SCLPbH	0	18.36	18.36	34.69^*^	Synergistic
SCHPbL	8.16	4.08	11.91	20.41^*^	Synergistic
SCHPbM	8.16	10.20	17.53	44.90^*^	Synergistic
SCHPbH	8.16	18.37	25.03	58.16^*^	Synergistic

According to a paired t-test (P<0.05 P<0.05), values in the ‘Observed Inhibition (O%)’ column marked with an asterisk (*) are significantly different from their corresponding ‘Expected Inhibition (E%)’ values.

*A. tauschii* leaf extracts differentially affected wheat seed germination and early seedling growth. The SCL resulted in positive Response Index (RI) values for all measured parameters, indicating a stimulatory effect (**Figure 2**). Conversely, SCH, Pb solutions (PbL, PbM, and PbH), and all combined treatments (SCLPbL, SCLPbM, SCLPbH, SCHPbL, SCHPbM, and SCHPbH) produced negative RI values for all indicators, signifying allelopathic inhibition. Notably, the inhibitory effect intensified with increasing Pb solution concentration, as evidenced by a significant decline in RI values for germination rate, seedling height, radicle length, and fresh weight (P< 0.05). This trend held regardless of whether Pb was applied alone or in combination with stem-leaf extracts, underscoring the dose-dependent role of Pb in overall allelopathic inhibition.

**Figure 3:**
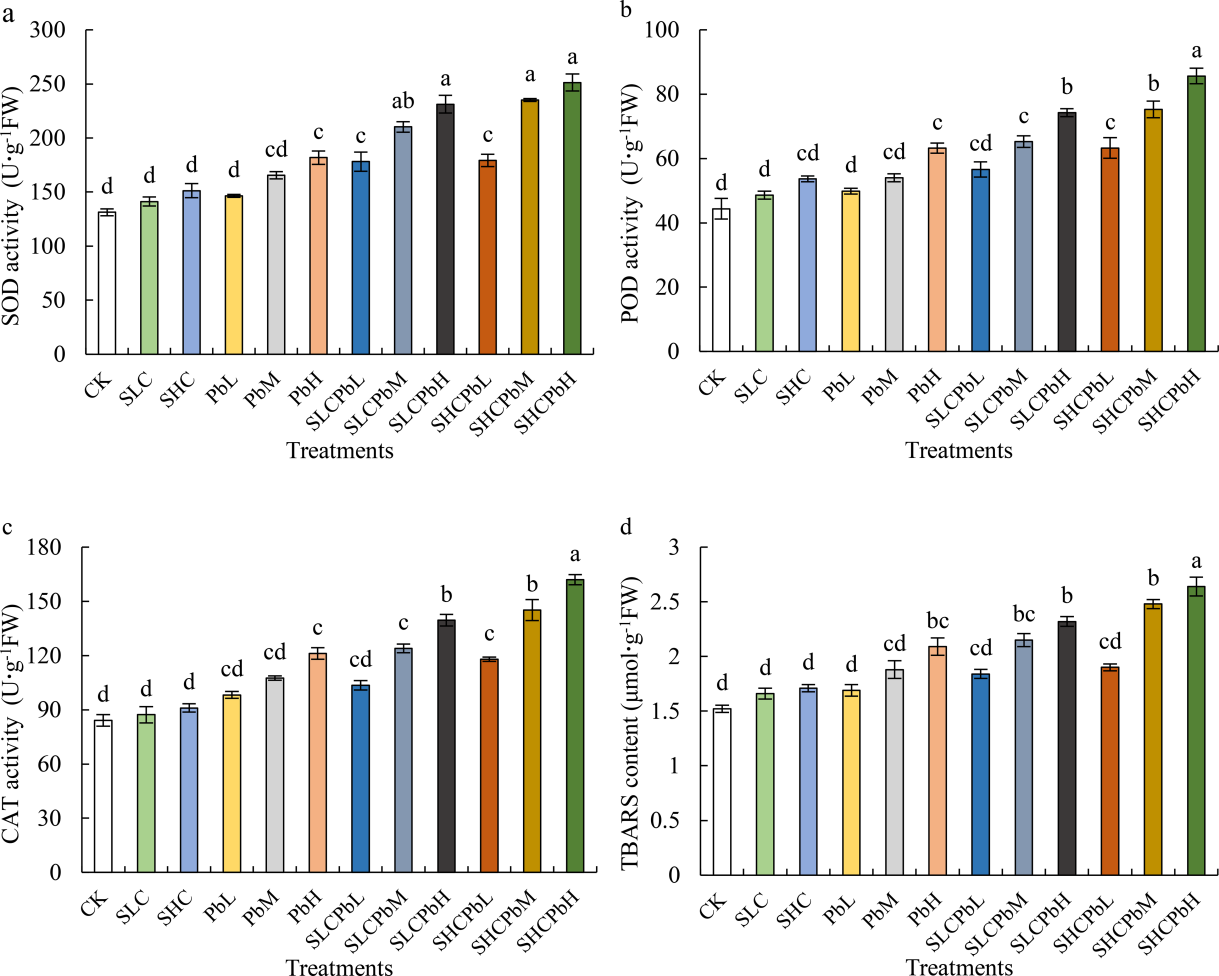
Activities of SOD, CAT, and POD; and TBARS content in wheat seeds in different treatment groups (see **Figure 1** for abbreviations). Bars (mean ± SE) with different lowercase letters indicate a significant difference (*P* < 0.05).

Compared with the control (CK), treatments involving SCL, SCH, and low- to medium-concentration lead solutions (PbL and PbM) had no significant effect on the activities of SOD, POD, and CAT in wheat seeds (**Figure 3**). However, the high-concentration Pb solution (PbH) and all combined treatments involving stem-leaf extracts and Pb solutions (SCLPbL, SCLPbM, SCLPbH, SCHPbL, SCHPbM, and SCHPbH) significantly increased the activities of SOD, POD, and CAT (*P* < 0.05). Specifically, SOD activity was significantly higher than in the CK treatment (*P* < 0.05) in the PbH, SCLPbL, SCLPbM, SCLPbH, SCHPbL, SCHPbM, and SCHPbH treatments. Similar significant increases were observed for POD and CAT activities across these treatment groups.

TBARS content in wheat seeds was not significantly affected by individual treatments with SCL, SCH, PbL, or PbM. However, the PbH treatment and all the combined treatments (SCLPbL, SCLPbM, SCLPbH, SCHPbL, SCHPbM and SCHPbH) significantly increased TBARS levels. Specifically, the PbH, SCLPbM, SCLPbH, SCHPbM, and SCHPbH treatments showed significant increases compared to the control group (*P* < 0.05). Of all the treatments, SCHPbH had the most pronounced effect, increasing SOD, POD, and CAT activities, as well as TBARS content, by 40.14%, 35.42%, 37.28% and 38.95% respectively, compared to the control (*P* < 0.05).

**Figure 4:**
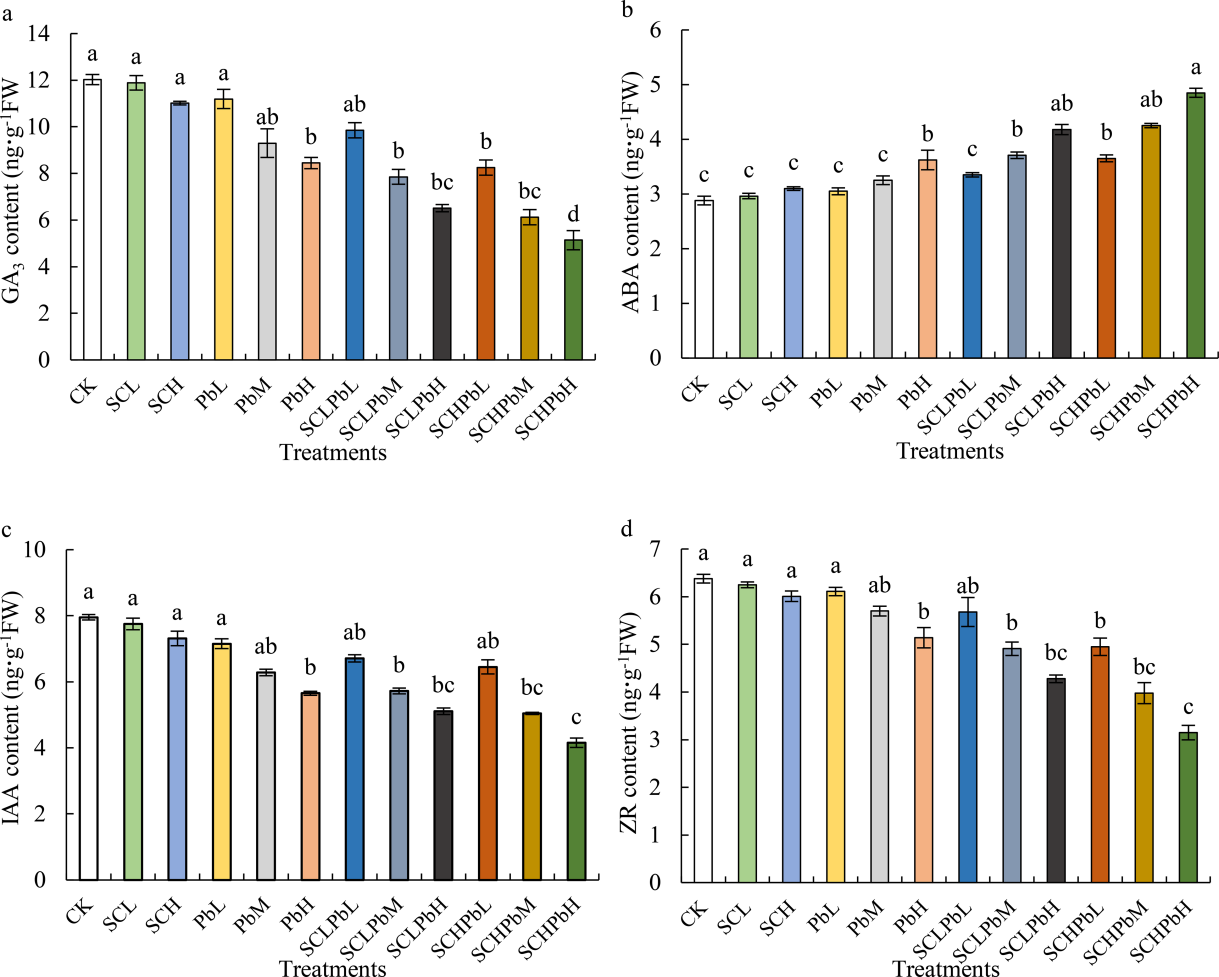
Phytohormone contents in wheat seeds in different treatment groups (see **Figure 1** for abbreviations). Bars (mean ± SE) with different lowercase letters indicate a significant difference (*P* < 0.05). **(a)** GA_3_, **(b)** ABA, **(c)** IAA, **(d)** ZR.

Compared to the CK, individual treatments with *A. tauschii* extract (SCL and SCH) or low-to-moderate Pb concentrations (PbL and PbM) did not significantly alter phytohormone levels in wheat seeds (**Figure 4**). However, a high Pb concentration (PbH) and all combined treatments (extract + Pb) significantly decreased GA_3_, IAA, and ZR content while increasing ABA content (*P* < 0.05). Specifically, GA_3_ and ZR contents were significantly reduced in the PbH, SCLPbM, SCLPbH, SCHPbL, SCHPbM, and SCHPbH treatments compared to the CK (*P* < 0.05). Similarly, these treatments significantly altered ABA and IAA content relative to the control (*P* < 0.05). The SCHPbH had the most pronounced effects, with GA_3_, IAA, and ZR contents decreased by 37.70%, 35.66%, and 36.36%, respectively, while ABA content increased by 40.58% compared to the CK (*P* < 0.05).

**Figure 5:**
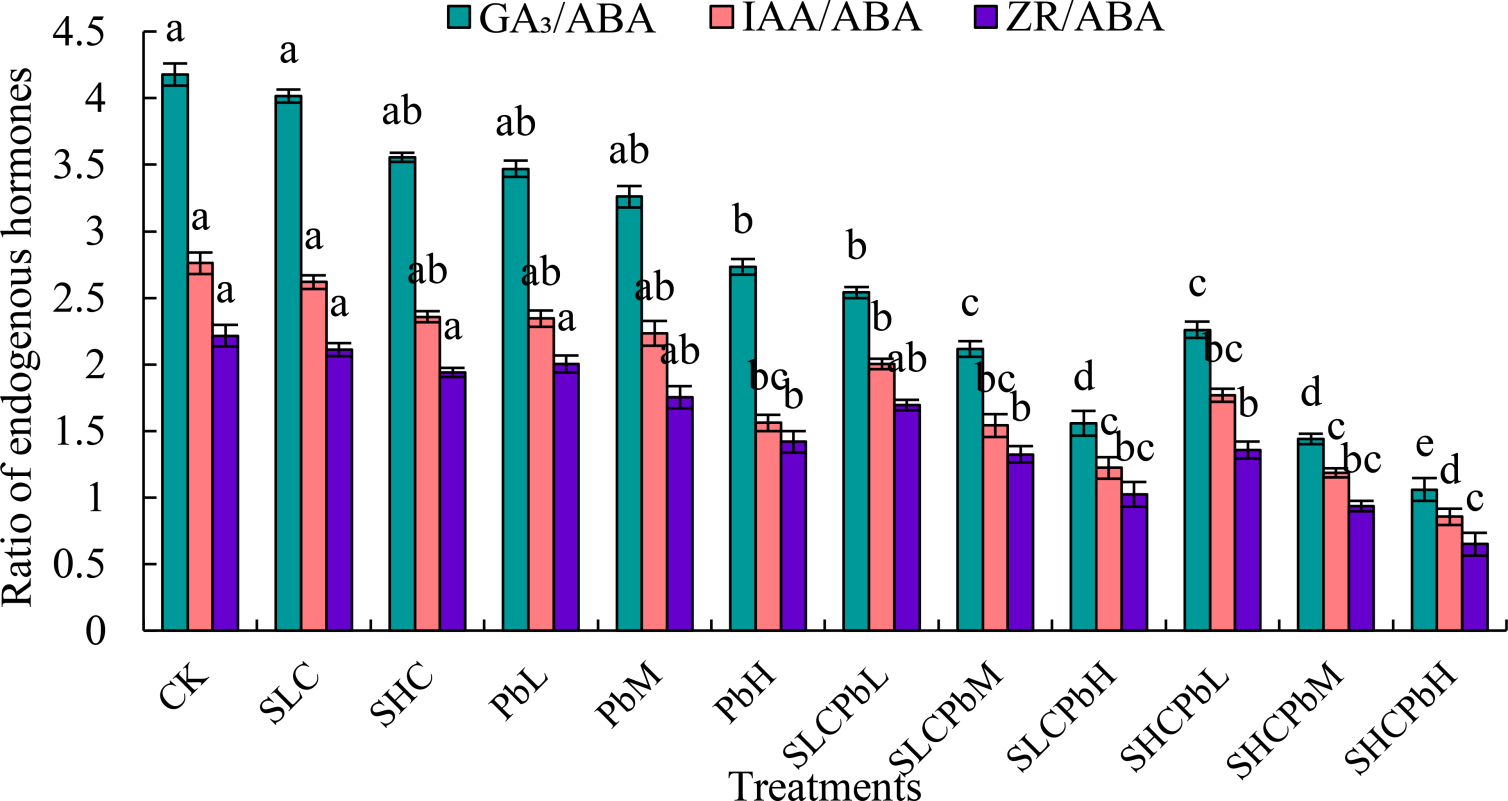
Ration of endogenous hormones in wheat seeds in different treatment groups (see **Figure 1** for abbreviations). Bars (mean ± SE) with different lowercase letters indicate a significant difference (*P* < 0.05).

Compared to CK, all of the tested treatments significantly decreased the GA_3_/ABA, IAA/ABA, and ZR/ABA ratios in wheat seeds (**Figure 5**). These treatments included stem-leaf extracts alone (SCL and SCH), Pb solutions alone (PbL, PbM, and PbH), and a combination of stem-leaf extracts and Pb treatments (SCLPbL, SCLPbM, SCLPbH, SCHPbL, SCHPbM, and SCHPbH). Specifically, the GA_3_/ABA and IAA/ABA ratios were significantly lower in the PbH, SCLPbL, SCLPbM, SCLPbH, SCHPbL, SCHPbM, and SCHPbH treatments than in the CK (*P* < 0.05). Similarly, the ZR/ABA ratio decreased significantly in the PbH, SCLPbM, SCLPbH, SCHPbL, SCHPbM, and SCHPbH treatments compared to the CK (*P* < 0.05). The SCHPbH treatment had the strongest inhibitory effect, resulting in reductions of 74.63%, 69.00%, and 70.68% in the GA_3_/ABA, IAA/ABA, and ZR/ABA ratios, respectively, compared to the CK (*P<* 0.05).

## Discussion

Invasive plants often use allelopathy to release bioactive compounds that suppress the growth of native species, facilitating population expansion and successful colonization (Kalske et al., 2022). These allelochemicals directly impact native species by reducing seed germination rates and inhibiting seedling development (Wang et al., 2018). Our study quantitatively demonstrates that the allelopathic effects of *A. tauschii* on wheat are significantly exacerbated by Pb contamination. Analysis using Colby’s method provided conclusive evidence of a synergistic interaction (**Table 2**), thereby fully supporting our first hypothesis (H1). This synergy explains why the combined effects of the treatments were far more severe than the sum of their individual effects.

### Allelopathic effects of *A. tauschii* leaf and stem extracts on wheat seed germination and early seedling growth under Pb Stress

SGerSGro are fundamental processes determining plant survival and community structure (Yuan et al., 2013). This research evaluated the allelopathic potential of *A. tauschii* stem and leaf extracts at different concentrations (SCL and SCH) on wheat seed germination rate, seedling height, radicle length, and fresh weight. Our findings indicate that individual applications of SCL and SCH did not significantly alter wheat seed germination and early seedling growth. Nevertheless, the calculated RI values suggest that low levels of *A. tauschii* invasion (i.e., lower extract concentrations) may exert negligible allelopathic effects on wheat. Significant allelopathic inhibition is therefore more likely to manifest under conditions of increased *A. tauschii* invasion intensity, corresponding to higher extract concentrations. This finding is consistent with reports on other invasive plants, including Canadian goldenrod (*S. canadensis*) (Ye et al., 2014; Lu et al., 2020), annual fleabane (*Erigeron annuus* L.), and Canadian fleabane (*Conyza canadensis* L.) (Lu et al., 2020). It has been proposed that this response may be associated with the release of low concentrations of allelochemicals by invasive species, which induce the production of reactive oxygen species at cell elongation sites and thereby stimulate growth in native plants (Duke et al., 2006; Prithiviraj et al., 2007). Widely recognized as a key adaptive strategy of plants to environmental change, this growth-stimulating effect is frequently interpreted as a hormone-like response (Agathokleous, 2018; Agathokleous et al., 2019).

HMs not only affect seed germination but also significantly influence seedling growth traits, including root length, shoot height, and fresh weight (Gao et al., 2014). Pb, in particular, is a non-essential element for plants and is particularly toxic to wheat, typically exerting a strong inhibitory effect on both germination and seedling development in a typically dose-dependent manner (Lamhamdi et al., 2011). However, some studies have reported that low concentrations of Pb can enhance wheat seed germination, whereas higher concentrations cause significant inhibition (Xia, 2018; Yang, 2018). The results of this study showed that treatment with a low Pb concentration (PbL) did not significantly affect wheat seed germination or early seedling growth, relative to the CK. By contrast, medium and high Pb concentrations (PbM and PbH) markedly reduced the germination rate, seedling height, radicle length, and fresh weight. Analysis of the relative index (RI) values confirmed that Pb exerted a dose-dependent inhibitory effect on wheat seed germination and early seedling development. These findings differ from those reported by Xia (2018) and Yang (2018), which may be due to variations in Pb tolerance among wheat cultivars.

With the rapid progression of industrialization and the excessive use of chemical fertilizers, pesticides, and livestock manure in agricultural production, Pb contamination in China’s agricultural soils has become increasingly severe. Under conditions of intensified HM pollution, the allelopathic effects of invasive species on SGerSGro of native plants may be altered or even amplified (Wei et al., 2020a). This aligns with our first hypothesis (H1) that Pb contamination synergistically enhances the phytotoxicity of *A. tauschii* toward wheat seedlings. This study demonstrated that the combined treatment of stem-leaf extract and Pb solution (SCLPbL, SCLPbM, SCLPbH, SCHPbL, SCHPbM, and SCHPbH) significantly inhibited wheat seed germination and early seedling growth. The RI values for all parameters were consistently below zero across all combined treatments. Additionally, the germination rate, seedling height, radicle length, and fresh weight were all significantly lower under the combined treatments than under the stem–leaf extract or Pb solution applied alone. These results are consistent with observations in *S. canadensis* (Wang et al., 2018) and may be explained by the synergistic stress resulting from the interaction between allelochemicals and Pb exposure or the enhanced production of allelochemicals by Pb in *A. tauschii*. Overall, the findings suggest that, under Pb-contaminated conditions, the inhibitory impact of *A. tauschii* on wheat seed germination and early seedling growth intensifies with increasing levels of infestation.

### Allelopathic effects of *A. tauschii* stem–leaf extracts on the physio-biochemical characteristics of wheat seed germination under Pb stress

The synergistic inhibition quantified by Colby’s method can be attributed to several interconnected physiological mechanisms (Andrade et al., 2019). Pb stress likely acts as a primary sensitising agent, compromising the cellular integrity and defence systems of wheat and rendering it more susceptible to the phytotoxins present in the *A. tauschii* extract. This ‘priming’ effect could facilitate the uptake and efficacy of allelochemicals, leading to an amplified disruptive impact on hormonal balance and oxidative homeostasis (Hayat et al., 2020). Our physiological and biochemical data strongly support this model, revealing how combined stress induces a severe oxidative burst and hormonal imbalance synergistically, which manifests as a dramatic decrease in the GA_3_/ABA ratio and a sharp increase in TBARS content.

Seed germination is a crucial initiating phase of the plant life cycle, orchestrated by enzymatic regulation. Sequential reactions mobilize seed storage reserves and support biosynthesis and expansion in the embryonic axis, providing the energy and precursor metabolites required for seedling establishment (Zhu et al., 2023). However, the intense metabolic activity accompanying germination elevates reactive oxygen species (ROS) production, which can impose oxidative stress if unchecked (Farooq et al., 2021). Maintaining cellular redox homeostasis, therefore, requires a dynamic balance between ROS generation and scavenging. This balance also mediates plant responses to environmental cues, including allelochemicals and HMs (Mittler et al., 2022). Upon exposure to allelochemicals or HMs, ROS can accumulate rapidly at the site of contact. In response, plants activate enzymatic antioxidant defences—including SOD, POD, and CAT—to detoxify excess ROS, limit cellular damage, and modulate subsequent growth and developmental processes (Fujita and Hasanuzzaman, 2022). This study demonstrated that stem-and-leaf extracts of *A. tauschii* (SCL, SCH), as well as low- and medium-concentration Pb treatments (PbL, PbM), did not significantly affect SOD, POD, or CAT activities in wheat seeds. These results may reflect intrinsic adaptive mechanisms in wheat and align with the observed levels of TBARS content. TBARS is a widely used indicator of membrane lipid peroxidation in plants; increases in TBARS reflect greater membrane injury and can compromise seed viability (Doria et al., 2019).

The synergistic phytotoxicity observed under combined Pb and *A. tauschii* extract treatments was mediated by severe disruption to wheat’s physiological and biochemical defence mechanisms, thus supporting our second hypothesis (H2). Specifically, although antioxidant enzyme activities (SOD, POD and CAT) increased significantly under high Pb and combined stress, this response was insufficient to counteract the oxidative burst. This led to significant cell membrane damage, as indicated by increased TBARS content. This highlights the compromised antioxidant defence system under combined stress. In our study, neither the high−Pb treatment (PbH) nor the low−concentration stem–leaf extract plus Pb treatment (SCLPbL) elicited significant increases in CAT or POD activity relative to their respective controls (*P* > 0.05), whereas SOD activity was more responsive. This pattern is consistent with the view that SOD functions as the primary enzymatic defence against oxidative stress (Kamran et al., 2021).

Seed germination is closely associated with dynamic changes in endogenous phytohormone levels. Multiple phytohormones coordinate the mobilization of stored reserves and the activation of biosynthetic pathways, thereby driving the germination process (Fu et al., 2024). GA_3_, for example, promotes germination by upregulating hydrolytic enzymes (e.g., α-amylase), accelerating the degradation and utilization of seed reserves (Shu et al., 2016). In contrast, ABA reinforces dormancy by antagonizing gibberellin signalling and modulating gene expression, thereby restraining germination programs (Zhao et al., 2024). The role of indole-3-acetic acid (IAA) appears context dependent: some studies report facilitation of dormancy release (Wang et al., 2020), whereas others find no significant association (Yang and Wang, 2012). Zeatin riboside (ZR) can counteract germination inhibitors and often shows a negative correlation with seed dormancy (Wu et al., 2015). This study revealed that, relative to treatments with stem–leaf extract alone (SCL, SCH) or with low- and medium-concentration Pb solutions (PbL, PbM), both high-concentration Pb treatment (PbH) and all combined stem–leaf extract plus Pb treatments (SCLPbL, SCLPbM, SCLPbH, SCHPbL, SCHPbM, SCHPbH) significantly decreased the levels of GA_3_, IAA, and ZR in wheat seeds (*P<* 0.05), while significantly increasing ABA content (*P* < 0.05). This indicates that treatment with high-concentration Pb solutions and stem-leaf extract solutions significantly inhibited wheat seed germination, which is consistent with the aforementioned measurements of the seed germination rate. Seed dormancy and germination are not determined by the absolute concentration of a single endogenous hormone; rather, they depend on the dynamic balance among multiple hormones, particularly the ratios of growth-promoting to inhibitory hormones (Shu et al., 2016). In this study, high-concentration Pb treatment (PbH) and combined treatments with stem–leaf extract and Pb (SCLPbL, SCLPbM, SCLPbH, SCHPbL, SCHPbM, SCHPbH) significantly decreased the GA_3_/ABA and IAA/ABA ratios in wheat seeds (*P* < 0.05). This disruption of hormone balance, including the significant decrease in GA_3_/ABA and IAA/ABA ratios under high Pb and combined treatments, directly supports H2 by demonstrating a compromised hormonal regulation crucial for germination. These reductions suggest that both high Pb exposure and combined extract–Pb treatments suppress wheat seed germination by simultaneously lowering growth-promoting hormones and enhancing the accumulation of inhibitory hormones.

Overall, our results indicate that SGerSGro are sensitive indicators of stress, with physiological and hormonal metrics serving as key tolerance markers. Furthermore, we demonstrate that Pb contamination amplifies the phytotoxicity of *A. tauschii* towards wheat by disrupting antioxidant defences and hormonal homeostasis. Understanding these mechanisms clarifies how abiotic stressors promote the invasiveness of allelopathic weeds and informs strategies for invasion management and agroecosystem restoration.

## Conclusion

In summary, Colby’s method has quantitatively confirmed a strictly synergistic interaction between *A. tauschii* extract and Pb contamination. This synergy markedly intensified the allelopathic effects on wheat, resulting in severe physiological disruption under the combined high-concentration treatment (SCHPbH). This treatment significantly impaired the antioxidant system (SOD, POD and CAT), induced severe membrane lipid peroxidation (as evidenced by a sharp increase in TBARS) and disrupted hormonal homeostasis by drastically reducing growth-promoting hormones (GA_3_, IAA and ZR), while increasing ABA. The consequent collapse of the GA_3_/ABA and IAA/ABA ratios critically compromised germination and early growth. Given the increasing prevalence of Pb in agricultural soils and the ongoing spread of *A. tauschii*, this interaction poses a serious threat to wheat productivity and agroecosystem stability.

## Data Availability

The original contributions presented in the study are included in the article/supplementary material. Further inquiries can be directed to the corresponding author.
